# Weak Responses to Auditory Feedback Perturbation during Articulation in Persons Who Stutter: Evidence for Abnormal Auditory-Motor Transformation

**DOI:** 10.1371/journal.pone.0041830

**Published:** 2012-07-23

**Authors:** Shanqing Cai, Deryk S. Beal, Satrajit S. Ghosh, Mark K. Tiede, Frank H. Guenther, Joseph S. Perkell

**Affiliations:** 1 Department of Speech, Language and Hearing Sciences, Boston University, Boston, Massachusetts, United States of America; 2 Speech Communication Group, Research Laboratory of Electronics, Massachusetts Institute of Technology, Cambridge, Massachusetts, United States of America; 3 Department of Brain and Cognitive Sciences and McGovern Institute for Brain Research, Massachusetts Institute of Technology, Cambridge, Massachusetts, United States of America; 4 Haskins Laboratories, New Haven, Connecticut, United States of America; 5 Department of Biomedical Engineering, Boston University, Boston, Massachusetts, United States of America; Northwestern University, United States of America

## Abstract

Previous empirical observations have led researchers to propose that auditory feedback (the auditory perception of self-produced sounds when speaking) functions abnormally in the speech motor systems of persons who stutter (PWS). Researchers have theorized that an important neural basis of stuttering is the aberrant integration of auditory information into incipient speech motor commands. Because of the circumstantial support for these hypotheses and the differences and contradictions between them, there is a need for carefully designed experiments that directly examine auditory-motor integration during speech production in PWS. In the current study, we used real-time manipulation of auditory feedback to directly investigate whether the speech motor system of PWS utilizes auditory feedback abnormally during articulation and to characterize potential deficits of this auditory-motor integration. Twenty-one PWS and 18 fluent control participants were recruited. Using a short-latency formant-perturbation system, we examined participants’ compensatory responses to unanticipated perturbation of auditory feedback of the first formant frequency during the production of the monophthong [ε]. The PWS showed compensatory responses that were qualitatively similar to the controls’ and had close-to-normal latencies (∼150 ms), but the magnitudes of their responses were substantially and significantly smaller than those of the control participants (by 47% on average, p<0.05). Measurements of auditory acuity indicate that the weaker-than-normal compensatory responses in PWS were not attributable to a deficit in low-level auditory processing. These findings are consistent with the hypothesis that stuttering is associated with functional defects in the inverse models responsible for the transformation from the domain of auditory targets and auditory error information into the domain of speech motor commands.

## Introduction

Developmental stuttering is a disorder of speech production characterized by frequent disruption of speech flow by involuntary repetitions and prolongations of speech sounds, as well as silent blocks. It affects approximately 1% of the adult population and typically has an onset in children between 3 and 5 years of age [1]. Despite recent advances in investigating the genetic (e.g., [2]) and neural (e.g., [3]–[8]) correlates of this disorder, the etiology and functional mechanisms of stuttering remain unclear.

Findings from behavioral and neurophysiological studies indicate that the interaction between auditory and speech motor functions may be critically involved in the mechanism of stuttering. Auditory feedback (AF), namely the auditory perception of one’s own speech during speech production, has been shown to play important roles in the learning and online control of speech in adults [9]–[15]. Recently, MacDonald et al. [16] showed that the speech motor adaptation in response to perturbation of AF can be found in children as young as 3 to 4 years of age, but not in two-year old toddlers. It is interesting to note that the age range of the onset of AF-mediated speech motor adaptation overlaps partially with the typical onset age range of developmental stuttering. Additional evidence for the involvement of AF in mechanisms of stuttering can be found in the conditions that lead to temporary improvements in the fluency of PWS. For example, manipulations of AF, such as noise masking, delaying, and frequency shifting, can significantly reduce dysfluency (e.g., [17]–[19]). In neuroimaging investigations of stuttering, several PET and functional MRI studies have reported weaker-than-normal activation of the left posterior superior temporal gyrus (pSTG) [20]–[24] or diminished functional connectivity between this area and other speech-related cortical areas [8] during speech in PWS. In fluent speaking control participants, the left pSTG is consistently activated during speech production and has been shown to be involved in the processing of AF [25], [26]. In addition, recent magnetoencephalography (MEG) studies have shown longer-than-normal latencies and abnormal inter-hemispheric latency asymmetry of the cortical responses (localized to the pSTG) to self-produced speech sounds in adults and children who stutter [27], [28]. Apart from functional abnormalities, MRI studies have shown structural abnormalities in the brain regions involved in speech-related auditory processing in PWS, including atypical inter-hemispheric asymmetry of the planum temporale (PT) [29]–[31].

Despite the confluence of evidence for a close relation between AF and stuttering, the exact nature of the abnormal auditory-motor interaction in stuttering remains unclear. There are several different ways that the influence of AF on speech production could be anomalous in PWS. First, PWS may have deficits in auditory processing that prevent them from perceiving their auditory errors as well as persons with fluent speech (PFS) can. Alternatively, they may hear auditory errors correctly but translate them incorrectly into motor corrective responses. This could take the form of an abnormal gain in the AF control system (i.e., a lower or higher than normal motor compensation to an auditory error) or increased variability in the motor response for a given auditory error. Finally, PWS may have normal auditory perception and AF control mechanisms (indicated by normal responses to feedback perturbation) but may not be as proficient at incorporating corrective motor commands into stored motor programs (or feedforward commands; cf. [32]) for speech sounds. In the current study we use an unexpected AF perturbation paradigm to begin to untangle these possibilities.

**Figure 1:**
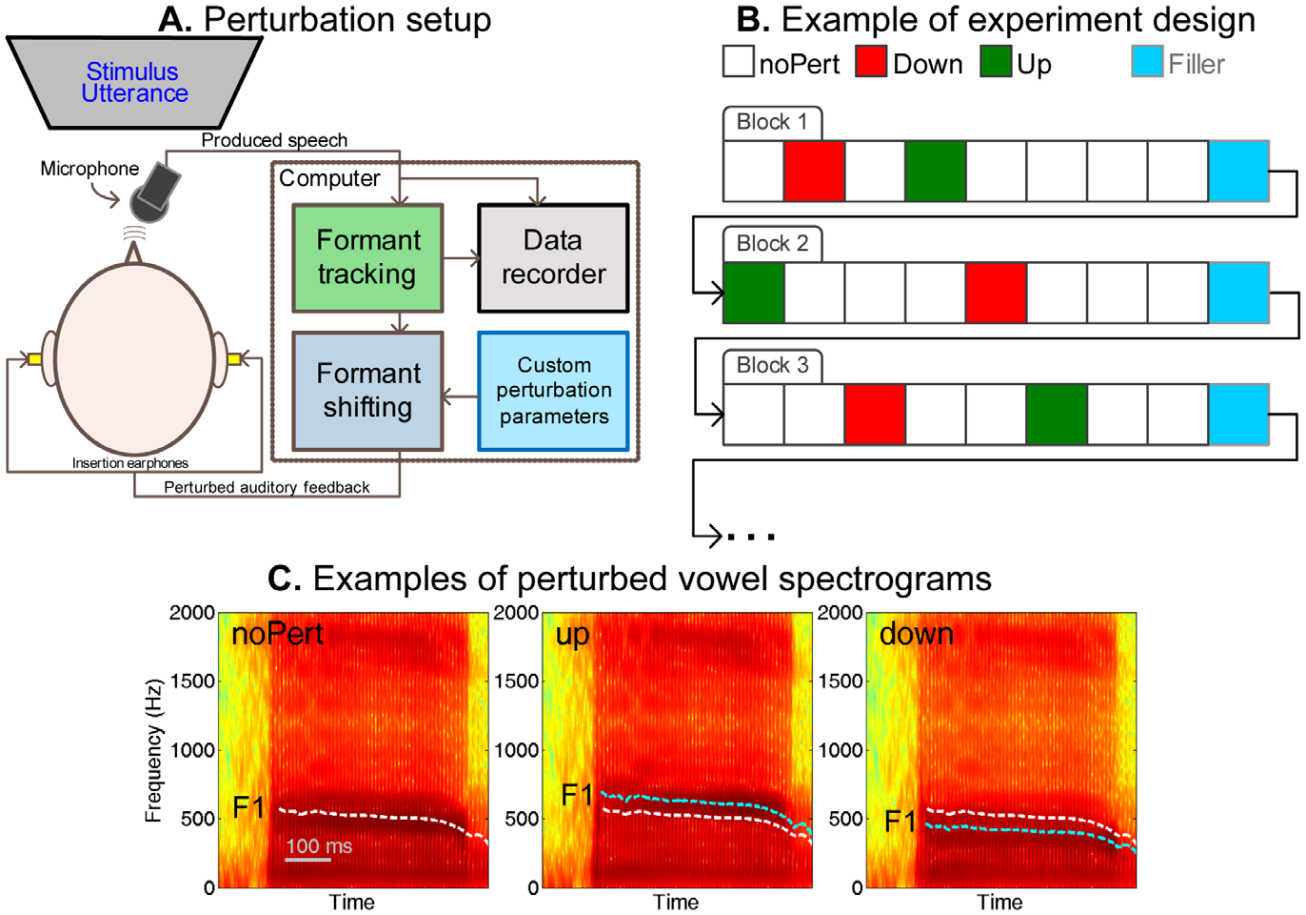
Design of the experiment. **A.** A schematic diagram of the setup used for AF perturbation during speech. **B.** A schematic showing an example of the ordering of the noPert (baseline), Down, and Up trials in Experiment 1. Note the rule that two perturbation trials were separated by at least one intervening noPert trial. **C.** Example spectrograms of the monophthong [ε] in the word “head”: the original (noPert) spectrogram (left), 20% Up shift (center), and 20% Down shift (right). The dashed white curves show the original tracked F1 trajectories; the dashed cyan curves display the perturbed F1 trajectories.

Real-time perturbation to AF of formant frequencies has been used in a number of prior studies to probe the role of AF in speech [12], [25]. Formants are resonance peaks in the spectrum of speech sounds that are determined by and thus reflect the positions of the articulators used in producing these sounds (e.g., see Fig. 1C). Under this type of online perturbation, normally fluent speakers show parameter-specific online articulatory adjustments in the direction opposite to that of the perturbation [12], [25]. We took this online perturbation approach in the current study. Specifically, we measured the auditory capabilities and corrective motor responses to unexpected perturbations to the first formant frequency (F1) of monophthongs (quasi-static vowels) of ongoing speech in PWS and a control group of persons who are fluent speakers to test the following hypotheses:

H1: PWS have a deficit in auditory perception that affects their ability to compensate for auditory perturbations, evidenced by a reduced ability to distinguish formant frequency differences in an auditory discrimination task.

H2: PWS have an abnormal gain in their AF control systems for speech, evidenced by smaller or larger than normal responses to AF perturbation.

H3: PWS have abnormal variability in their motor responses to AF errors, as evidenced by greater variability (across trials) than PFS in their motor responses to auditory perturbations.

## Materials and Methods

### 2.1. Participants

All participants were right-handed as measured via the Edinburgh Handedness Inventory [33] and had a negative history of medical or developmental disorders, except for developmental stuttering in the PWS group. All participants were native speakers of American English. Twenty-one PWS (16 male, 5 female; age range: 18–47; median: 25) were recruited through referrals from speech-language pathologists in the Boston area and advertisement on Internet websites and blogs. A certified speech-language pathologist (D.S.B., second author) screened all the PWS participants to confirm the diagnosis of persistent developmental stuttering and the absence of comorbid speech, language or hearing disorders. The Stuttering Severity Instrument −4^th^ Edition (SSI-4; [34]) was administered to all PWS participants to quantify the severity of their overt stuttering characteristics. The SSI-4 scores of the PWS participants ranged from 13 to 43 (median = 25; inter-quartile range = 11.25), covering a range of severity from mild to very severe. Eighteen PFS (14 male, 4 female) were recruited as control participants. Their ages ranged from 19 to 43 (median: 25) and did not differ significantly from the age range of the PWS group (p>0.94, two-tailed Wilcoxon rank-sum test). The participants gave written informed consent under the protocols approved by the Massachusetts Institute of Technology Committee on the Use of Humans as Experimental Subjects (protocol number: 1003003787).

### 2.2. Procedure

Figure 1A contains a schematic diagram of the experimental setup used in the current study. Participants were seated comfortably in front of a computer monitor, which displayed words or sentences to be read aloud, together with additional experimental prompts and instructions. Audapter [35], custom in-house MEX-based software written in Microsoft Visual C++ and executed under MATLAB, was used to track and shift the formant frequencies in real time with a latency of 11 ms. The speech signals, sometimes with shifted formants, were played back to the participant through a pair of insertion earphones (Aearo Technologies). The participant’s produced speech signals and formant trajectories were recorded at sampling rates of 12000 and 750 Hz, respectively, for subsequent analysis.

Participants were instructed to produce the two words “head” and “pet”. Each word contained the monophthong [ε], embedded in a monosyllabic consonant-vowel-consonant (CVC) syllable. Before the beginning of the data gathering phase, the participants were trained to produce the words within a medium range of vocal intensity (74–84 dB SPL as measured by a microphone secured at 10 cm from the subject’s mouth) and a medium range of vowel duration (300–500 ms). Visual feedback regarding their success or failure in achieving these ranges was provided during the training phase of the experiment. In the data gathering phase, the participants were instructed to try to stay within the learned intensity and duration ranges. Warning messages were given on the screen if they exceeded either of these ranges. This procedure ensured an approximate consistency of intensity and speaking rate across trials, conditions, participants, and subject groups.

The data-gathering phase contained 160 trials, arranged into 20 blocks of eight words. Each block consisted of four trials of the word “head” and four trials of the word “pet”, in pseudo-randomized order. As the example in Fig. 1B shows, two of the eight trials in each block were selected to contain perturbation of F1: one of them incorporated the 20% upward (“Up”) perturbation and the other the 20% downward (“Down”) perturbation of F1, similar to the perturbations used by Tourville et al. [25]. Figure 1C shows an example spectrogram of the utterance “head”, and the Down- and Up-perturbed versions of this spectrogram. In the remaining six trials, the participants received AF that contained no perturbation to the formants. These trials will be referred to as the *no-perturbation* (*noPert*) or *baseline* trials. The order of the noPert and perturbed trials was pseudo-randomized, with the only constraint that trials with perturbation did not occur in two consecutive trials in a block (See the example in Fig. 1B). A “filler” trial, consisting of a sentence randomly drawn from the IEEE sentence pool [36], was inserted between two blocks, to increase stimulus variety and to reduce the boredom experienced by the participants.

### 2.3. Perturbation of Formant Frequencies

The Audapter software was used for manipulating the AF of F1. This software has been described in detail previously [13], [14], [35]. Briefly, the microphone signal was digitized at a sample frequency of 48000 Hz and downsampled by a factor of 4 to 12000 Hz for real-time processing. An autoregressive linear predictive coding algorithm, followed by a dynamic-programming tracking algorithm [37], was used to estimate the formant frequencies in near-real time. The tracked formant frequencies were then mapped to new, shifted values. In this experiment, fixed-ratio (+20% or −20%) shifting of F1 was used. Once the shifted formant frequencies were determined, a pole-substituting digital filter served to bring the formant resonance peaks from their original values to the new ones. The total latency of the artificial AF loop was 11 ms.

### 2.4. Data Analysis

The first author (S.C.), blinded from the perturbation conditions of all trials, manually examined the audio recordings. Trials that contained speech errors, dysfluencies, or gross formant tracking errors were discarded from further analysis. Discarded trials amounted to 0.25% of the trials from the PWS and 0.17% of the trials from the PFS. Only a very small fraction (0.063%) of the trials from the PWS group contained audible occurrences of dysfluency. The most likely factors responsible for this low dysfluency rate were the simplicity of the speaking material (isolated single words) and the relatively slow speaking rate required in this experiment were the potential factors contributing to the relatively low level of dysfluency shown by the PWS in the current experiment.

The formant trajectories were smoothed with 28-ms Hamming windows. To analyze the F1 produced by the participants, the F1 trajectories were aligned from the time of vowel onset (as determined by signal root-mean-square intensity thresholding) and averaged across the trials frame-by-frame for each condition, giving rise to three average trajectories from each subject (noPert, Down, and Up). Data from the first 300 ms (i.e., the lower limit of the vowel duration target range) were included in this averaging. In order to ensure that the number of individual trials included in this averaging was uniform from the onset to the offset of the average trajectories, we discarded the trials with vowel durations shorter than 300 ms, i.e., trials in which the participants produced vowels that were shorter than required. The percentage of trials discarded due to failure to meet this minimum vowel-length criterion were 13.7% and 10.1% in the PWS and PFS groups, respectively, which did not differ significantly (p>0.26, two-tailed Wilcoxon rank-sum test). The average formant trajectories were then submitted to group-level statistical analysis.

To analyze the production measures, we used repeated measures analysis of variance (RM-ANOVA), with perturbation condition (noPert, F1-Up and F1-Down) as the within-subject factor. Violations of the sphericity assumption [38] of RM-ANOVA were corrected with the Huyhn-Feldt correction. *Post hoc* comparisons followed the finding of significant main effects or interactions in the RM-ANOVA. Two-tailed t-tests were used for between-group comparisons. A significance threshold of α = 0.05 was used. Corrections for multiple comparisons were done with the False Discovery Rate (FDR, [39]).

### 2.5. Measurement of Participants’ Auditory Acuity to Vowel F1 Change

Following the afore-described AF perturbation experiment, within the same two-hour experimental session, a psychophysical experiment was conducted to measure the auditory perceptual acuity of the participants to changes in the F1 of the vowel [ε]. This perceptual test utilized the adaptive staircase procedure (also known as the adaptive up-down procedure, [40]).

Our implementation of the adaptive staircase involved a series of two-alternative-forced-choice trials. In each trial, three vowel sounds were played in succession, with the second or the third one different from the first (standard) sound, while the remaining one was identical to the standard. Therefore there were two possible scenarios for each trial: “ABA”, i.e., second sound different from the standard, and “AAB”, i.e., the third different from the standard. The ordering of the two scenarios was randomly generated with equal probabilities (0.5).

The task of the participant was to judge whether the second or the third sound was different from the standard. At the beginning of this experiment, the participants were informed verbally that the purpose of the test was to determine the smallest difference between two vowel sounds that they could detect. They were instructed to listen carefully, especially when the difference between the standard and the non-standard was small. Participants were encouraged to make their best guesses if unsure about the correct choices. After each trial, the participants were provided visual feedback regarding the correctness of their choices, to encourage consistent performance throughout the course of the test.

To ensure that the result of the perceptual test was generalizable to the AF perturbation condition, the standard sound (A) was a synthesized steady-state vowel of which the first and second formant frequencies (F1 and F2) were equal to the most typical vowel [ε] produced by the subject in the noPert condition in the preceding AF perturbation-production experiment. The most typical trial was determined by plotting the F1 and F2 of the vowels in the 2-dimensional formant space and choosing the one that lay closest to the center of gravity (2-dimensional arithmetic mean) of the data set. The duration of each vowel sound was 300 ms. A 500-ms gap was inserted between each adjacent pair of vowels. Hence the stimulus used in each trial had a total duration of 1900 ms. The F0 of the vowel was equal to the arithmetic mean F0 of the vowel [ε] produced by the participant in the unperturbed condition of the AF perturbation experiment. The standard and nonstandard vowels were synthesized with a MATLAB implementation of the Klatt synthesizer [41].

In each run of the adaptive staircase procedure, the B (i.e., non-standard) stimulus had a F1 higher than the A stimulus (standard). The amount of the F1 difference was initially set to the magnitude of the perturbation used in the AF perturbation experiment (20%). A two-down-one-up paradigm [42] was used. If the participant made correct choices in two consecutive trials, the amount of the A–B difference was reduced. Conversely, the A–B difference was increased if a wrong choice was made. Each change in the sign of the increment of the A–B difference constituted a *turn*. The absolute amount of the increment of the A–B difference also changed at each turn. The change amount was initially 25% of the original A–B difference (i.e., 5% of the perturbation magnitude used in the production experiment), and decreased according to a harmonic series of the number of turns (1/n_turns_). Each staircase was terminated as soon as the sixth turn was reached. The amount of A–B difference at the end of each run was determined as the just noticeable different (JND) of that staircase. Each participant was administered six runs, with a 3–4 minute break between the third and fourth. The arithmetic mean of the JNDs from the last four runs was determined as the participant’s JND.

## Results

Table 1 summarizes the average vowel duration and levels produced by the PFS controls and PWS under the noPert, Down and Up conditions. A two-way mixed ANOVA with vowel duration as the dependent measure yielded no significant main effect of participant group (F_1,37_ = 0.028; p>0.86), nor any significant main effect of perturbation condition (F_2,74_ = 1.848; p>0.16). Similarly, there was no significant main effect of group (F_1,37_ = 0.220, p>0.65) or perturbation condition (F_2,74_ = 0.328; p>0.72) on vowel level.

**Table 1:** Summary of the vowel durations and levels produced under the three perturbation conditions (noPert, Down and Up) by the PFS (control) and PWS participants.

	Mean vowel duration (±1 SEM, ms)			Mean vowel level (±1 SEM, dB SPL)		
	*noPert*	*Down*	*Up*	*noPert*	*Down*	*Up*
**PWS**	386.3±8.3	384.0±7.3	390.3±9.3	77.70±0.39	77.76±0.42	77.70±0.40
**PFS**	381.8±8.7	383.7±10.5	388.9±10.3	78.07±0.36	77.90±0.36	77.96±0.34

The quantities shown are mean ±1 standard error of the mean (SEM) across subjects in each group.

The Down and Up F1 perturbations to the AF used in the experiments were based on fixed ratios of 20%. Under the Down perturbation, the average absolute perturbation magnitudes were 115.6±3.4 and 119.6±2.9 (mean±1 SEM) Hz in the PWS and PFS, which did not differ significantly (p>0.43, two-tailed t-test). Similarly, there was no significant difference in the absolute magnitude of the perturbations in the PWS (113.6±3.5 Hz) and the PFS (116.7±3.1 Hz) (p>0.5) under the Up perturbation.

**Figure 2:**
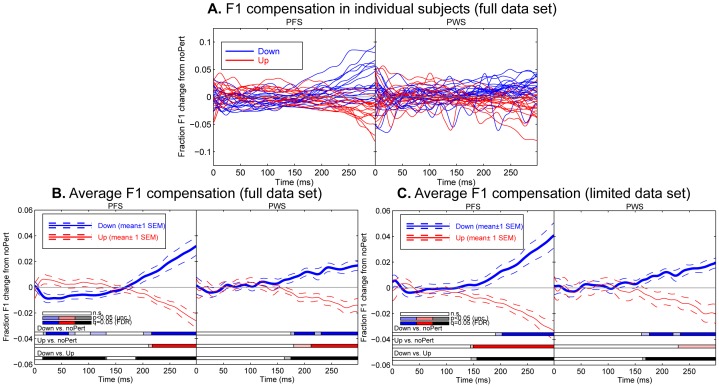
Compensatory adjustments of produced F1 trajectories under the Down and Up perturbations in PWS and PFS participants. **A.** Individual participants’ F1 trajectory changes from the noPert baseline, plotted as a function of time since vowel onset. Each blue (red) curve shows the data from the Down (Up) condition of one subject. The left and right parts of this plot show data of the fluent control (PFS) and PWS groups, respectively. This panel is based on the full data set (see text for details). **B.** Averages drawn from the same data as shown in Panel A. Solid curves: average F1 trajectory difference between the perturbed and noPert conditions, across all 18 PFS (left) and 21 PWS (right); dashed curves: mean±1 SEM. The three horizontal bars on the bottom of this panel indicate significant differences under three comparisons as functions of time. From top to bottom: Down vs. noPert, Up vs. noPert, and Down vs. Up. In each bar, the lighter color (lighter blue, lighter red, or lighter gray) indicates significance at an uncorrected threshold of p<0.05. The darker color (e.g., darker blue, darker red, or black) indicates significance at a corrected level of FDR  = 0.05. **C.** Same format as B, but with average F1 change trajectories computed based on the limited data set (see text for details).

Both groups of participants showed statistically significant compensatory responses to the perturbations of the AF of F1 during the production of the monophthong [ε] embedded in the CVC words “head” and “pet”. In Figure 2A, each red curve shows the difference between the average F1 trajectories produced under the Down and noPert conditions by a PFS control subject; similarly, each blue curve shows the difference between the average F1 trajectories produced under the Up and baseline conditions. As can be seen in this panel, there was considerable between-subject variability in their responses to the AF perturbations. However, the group-average responses (Fig. 2B) showed a systematic pattern of change of F1 in the productions in directions opposite to the perturbations, i.e., a gradual decrease under the Up perturbation and a gradual increase under the Down perturbation. Frame-by-frame t-tests were used to delineate the intervals in which these deviations from baseline were statistically significant at the group level. The light red parts of the horizontal bar in Fig. 2B indicate time intervals in which the difference between the F1 trajectories produced under the noPert and Up conditions reached statistical significance. Similarly, the light blue parts of the horizontal bar in the same panel indicate intervals in which the produced F1 trajectories under the noPert and Down conditions reached statistical significance (p<0.05 uncorrected, two-tailed t-test). In both horizontal bars, the darker-colored parts indicate the time intervals in which statistical significance was reached on a corrected level (FDR = 0.05). As can be seen from these bars, significant deviations from baseline commenced approximately 150–200 ms following the onset of the vowel (the onset of the perturbation). The magnitude of the compensation increased with time, and was approximately 3% (i.e., ∼15% of the perturbation) in the PFS group and 1.5% (i.e., ∼7.5% of the perturbation) in the PWS group at 300 ms following perturbation onset.

A seemingly puzzling aspect of the result from the PFS group is the significant deviations from the noPert baseline in the participants’ F1 productions in the same directions as the perturbations. These deviations can be seen in the first 100 ms following the onset of the perturbation (see the left part of Fig. 2B). These deviations reflected cross-trial adaptation similar to that shown in previous AF perturbation experiments that used sustained auditory perturbations (e.g., [11], [13], [43]–[45]), which demonstrated offline updating (i.e., adaptation) of the motor programs for the production of vowels. Due to the block-by-block randomized organization of the baseline, a perturbation trial always followed another perturbation trial of the opposite type, if it followed any perturbation trial in the same block (see Fig. 1B and the first sub-section of the Materials and Methods section). As a result, if a perturbation trial is preceded closely by another perturbation trial in the same block, the early part of the subject’s production in this trial may contain an adaptation response to the perturbation in the previous perturbation trial, which may be misrecognized as an apparent “early following” response to the perturbation in the same trial. Since such adaptive updating after-effects tend to decay during unperturbed productions (e.g., [13], [44]), a perturbation trial separated from the preceding perturbation trial by a larger number of baseline trials should show a weaker apparent early-following response of this type.

Consistent with this reasoning, when we included only the perturbation trials that were either preceded by no perturbation trial in the same block (e.g., the Down trials in Blocks 1 and 3 and the Up trial in Block 2 of the example in Fig. 1B) or separated from the preceding perturbation trial in the same block by at least three trials (e.g., the Down trial in Block 2 of the example in Fig. 1B), the apparent early following response disappeared (Fig. 2C). We will refer to this subset of data as the *limited data set*. It needs to be pointed out that the cross-trial adaptation effects were present not only in the Down and Up trials, but also in the noPert trials preceded closely by perturbation trials. However, since the noPert trials were preceded by Down and Up trials with equal probability, owing to the randomization of trial order, and because of the symmetry of the adaptation between the Down and Up directions, the cross-trial effects tended strongly to cancel out when all noPert trials were included to form the baseline condition.

**Figure 3:**
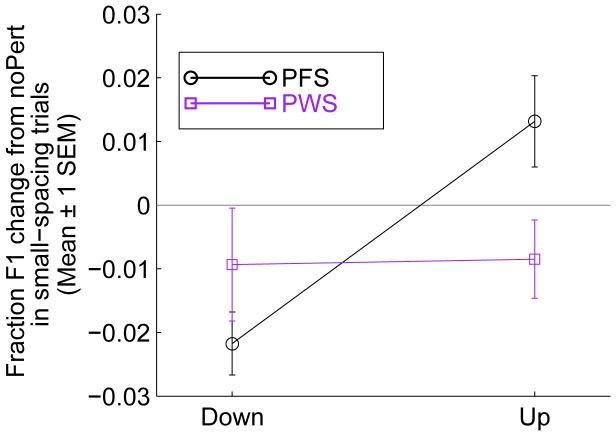
Different cross-trial adaptation responses in the PWS and PFS groups. The data used in generating this figure included Down and Up trials that were separated from the preceding perturbation trial (of the opposite type) in the same block by two or fewer trials, i.e., the small-spacing trials (see text for details). The formant frequency values in the first 50 ms of the vowel were averaged to generate the displayed results. Note the existence of the cross-trial adaptation effects, as shown by the large changes from the noPert condition, in the PFS group, and the lack thereof in the PWS group.

Interestingly, this cross-trial adaptation effect was not as pronounced in the PWS group as in the PFS group. This can be seen clearly by comparing the left part of Fig. 2B with the right part, in which the F1 changes in the first 100 ms were small and not significantly different from zero. To investigate the statistical significance of this between-group difference in cross-trial adaptation, we computed the average F1 changes from the no-perturbation baseline in the first 50 ms following the onset of the perturbation in the perturbation trials that were separated from the same-block preceding perturbation trials by two or fewer trials. The cross-trial adaptive response in the PFS group can be clearly seen in the black curve of Fig. 3: these changes were in the same directions as the perturbations, and as mentioned above, may be mistaken as “early following responses”. However, as can be seen from the purple curve of the same figure, these changes were smaller in absolute value and not significantly different from zero in the PWS group. We performed a two-way mixed ANOVA with the between-subject factor GROUP, which took the values of [PWS, PFS], and the within-subject factor SHIFT, which took the two levels [Down and Up]. The result of the ANOVA indicated a significant GROUP×SHIFT interaction (F_1,37_ = 5.68, p = 0.022), as well as a significant main effect by SHIFT (F_1,37_ = 5.93, p = 0.020). These results provide statistical confirmation of the observation that the cross-trial adaptation was weaker in the PWS than in PFS.

As Panels B and C of Fig. 2 show, the PWS showed compensatory responses that were qualitatively similar to those of the PFS: on average, the F1 trajectories in the subject’s productions deviated from the baseline values in directions opposite to the Up and Down perturbations. These compensatory F1 changes became significant at approximately 150 ms following perturbation onset. The same conclusion can be reached independent of whether the full (Fig. 2B) or limited (Fig. 2C) data set is examined. However, owing to the small size of the compensatory F1 corrections under the Up perturbation, significant F1 changes at the corrected level were reached only under the Down perturbation for the PWS. Comparing the data from the PWS and PFS in Fig. 2B, it can be seen that the magnitude of the compensatory responses were appreciably smaller in the PWS group than in the PFS group. The same conclusion can be drawn if the limited data set is considered (comparing Fig. 2C).

**Figure 4:**
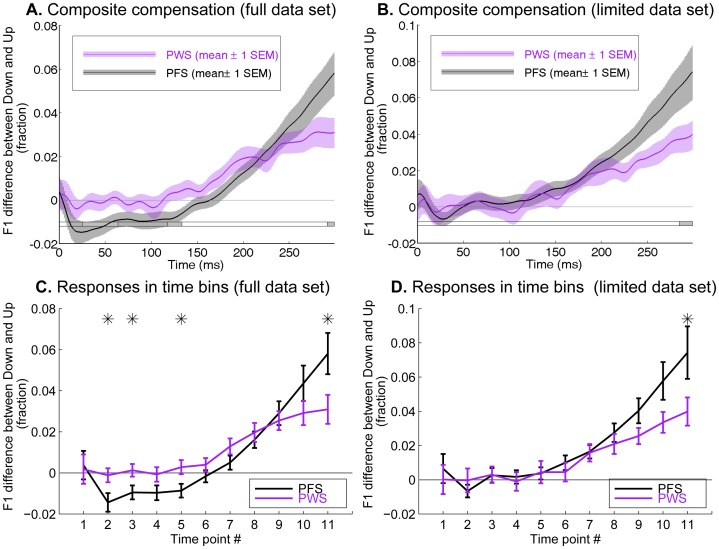
Average composite response curves from the PWS (purple) and PFS (black) groups. **A** and **B**: The composite compensation curves were computed by subtracting the F1 change profile under the Up perturbation from the F1 change profile under the Down perturbation. The horizontal bars below indicate significance of the difference between the two groups as a function of time (two-sample t-test, two-tailed). Gray: significance on the uncorrected level of p<0.05. Panels A and B illustrate the results from the full and limited data sets, respectively. Notice that the scales of the ordinates of Panels A and B are different. **C** and **D**: the composite response curves shown on a coarser time scale than in A and B. Eleven equally spaced time points were placed between 0 and 300 ms following vowel onset (30-ms separations). The error bars in these two panels show ±1 SEM. The asterisks at the top of the figure indicate time bins in which the difference between the PWS and PFS groups were statistically significant according to *post hoc* t-tests that followed the finding of significant GROUP×TPT interaction in the mixed ANOVA (see text for details). Results in C and D are based on the full and limited data sets, respectively.

To examine the statistical significance of the difference in magnitude of the compensatory responses between PWS and PFS, we computed the *composite response curve* for each participant by subtracting the Up response (e.g., red curves in Fig. 2B and C) from the Down responses (e.g., blue curves in Fig. 2B and C). This approach to reducing the dimensionality of the data was justified by the fact that the compensatory F1 corrections were largely symmetrical with respect to the perturbation directions in both the subject groups. Fig. 4A shows the average composite response curves in the PWS and PFS with the purple and black curves, respectively, computed on the full data set. Figure 4B showed the same average composite curves computed on the limited data set. It can be seen that regardless of whether the full or the limited data set was used, the magnitude of the composite response curves was smaller by approximately 47% in the PWS than in PFS at 300 ms following vowel onset.

To systematically analyze the statistical significance of the compensatory F1 changes and the between-group difference in the compensation magnitude, we performed a mixed analysis of variance (ANOVA). The dependent variable of the ANOVA was the Down-Up contrasts in the produced F1s of the participants, i.e., values in the composite response curves. These F1 contrasts were computed on 11 equally spaced time points between 0 and 300 ms following vowel onset. Note that the separation between adjacent time points was 30 ms, greater than the size of the smoothing Hamming window (28 ms), hence they did not cause correlations in the error terms. The 300-ms time limit was chosen because it was the lower bound of the vowel-duration range the participants were instructed to achieve. The two independent variables that entered the ANOVA were 1) GROUP, a between-subject factor, with two levels (PWS, PFS), and 2) time point (TPT), a within-subject factor, with the 11 levels that correspond to the above-mentioned eleven time points. Fig. 4C and D show the interval-averaged F1 compensation curves, under the full and limited data sets, respectively.

In this ANOVA, we were primarily interested in the main effect of TPT and the interaction between GROUP and TPT. The TPT main effect evaluates the significance of the compensatory F1 production changes when the data are collapsed across the PWS and PFS, whereas the GROUP×TPT interaction constitutes a test of the between-group difference in the trends of F1 change with time, i.e., magnitude of the compensatory responses.

The TPT main effect was highly significant regardless of the data set used (full data set: F_10,370_ = 37.6, p<1×10^−12^; limited data set: F_10,370_ = 30.7, p<1×10^−12^), clearly indicating the significance of the online compensatory adjustments of F1 in response to the AF perturbations when the data were collapsed across the two groups of subjects. In addition, the GROUP×TPT interaction reached significance for both data sets (full data set: F_10,370_ = 4.44, p = 0.006; limited data set: F_10,370_ = 2.729, p = 0.049, both with Huyhn-Feldt correction). In the *post hoc* comparison following the ANOVA with Tukey’s least significant difference (LSD) approach, the between-group difference in the latest two average intervals (270 and 300 ms following vowel onset) reached statistical significance under the limited data set, which confirms our informal observation earlier of the weaker-than-normal F1 compensation in PWS compared to the PFS responses (Fig. 4D). The *post hoc* comparisons for the full data set reached significance in the latest time point (300 ms), as well as in several earlier ones (before 150 ms from vowel onset), the latter of which confirmed again the significance of the weaker-than-normal between-trial adaptation in PWS than in PFS.

Consistent with previous findings (e.g., [9], [12]), compensatory responses to the auditory feedback could not be observed in all perturbation trials, despite the statistically significant compensation in the group-average data (Fig. 4). To characterize the between-trial variability in the responses and how it differed between PWS and PFS, we categorized the perturbation (Down and Up) trials into three categories: a. compensating, b. unresponsive and c. following. The average F1 in the last 50 ms of the [0, 300]-ms time interval following vowel onset was computed in each trial, and referred to as the F1_end_. The mean and standard deviations (SD) of the F1_end_ s under the noPert condition was computed for each subject. For each perturbation trial, if its F1_end_ deviated by more than one SD from the mean in the direction opposite to the perturbation, it was categorized as compensating; if its F1_end_ deviated by more than one SD from the mean in the same direction as the perturbation, it was categorized as following; otherwise the unresponsive category applied. Only the limited data set was used in this analysis.

As Table 2 summarizes, under the above criterion, the proportions of compensating responses were small (<30%), in both the PWS and PFS groups. These proportions were smaller compared to previous findings based on pitch perturbation (e.g., [9], [46]), which may be due to differences in pitch and articulatory control and/or due to the relatively short analysis window (300 ms) used in the current study. But these proportions were significantly greater than what would be expected if there were no differences in the distribution of F1_end_ between the noPert and perturbation conditions (15.9%; PFS: p = 0.00016, PWS; p = 0.0037; one-sample two-tailed t-test). The average proportion of compensating responses was slightly lower in the PWS than in the PFS, but this difference was not significant (p = 0.152, two-tailed t-test). On average, the PWS group showed a greater proportion of trials in the unresponsive category compared to the PFS, but this difference only approached significance (p = 0.086).

**Table 2:** Proportions of compensating, unresponsive and following responses under the Down and Up perturbations in the two groups of subjects.

	PFS	PWS	*p-value from t-test*
	(mean±1 SEM)	(mean±1 SEM)	(two-tailed)
**Compensating**	26.7%±2.3%	22.4%±2.0%	0.152
**Unresponsive**	63.7%±2.1%	68.6%±1.8%	0.086
**Following**	9.5%±1.5%	9.1%±1.5%	0.821

See text for details on the criteria of the three response categories.

To examine whether there was any systematic relationship between compensation magnitude and stuttering severity in the PWS group, we performed parametric and non-parametric correlational analyses between the Down-Up F1 fraction difference at 300 ms following vowel onset and the SSI-4 composite score across the PWS. No significant correlation was found, either under a linear Pearson product moment correlation (full data set: R^2^ = 0.00078, p = 0.70; limited data set: R^2^ = 0.00086, p = 0.90) or under a Spearman’s rho correlation (full data set: ρ = 0.080; p = 0.73; limited data set: ρ = 0.063; p = 0.79). When the sub-scores of SSI-4, including the frequency, duration, and concomitants scores, were correlated with the compensation magnitude, no significant correlations were found, either (full data set: R^2^ = 0.018, 0.015, 0.039 and p = 0.56, 0.59, 0.39; limited data set: R^2^ = 0.0040, 0.016, 0.057 and p = 0.78, 0.58, 0.30 for frequency, duration and concomitants scores, respectively).

**Figure 5:**
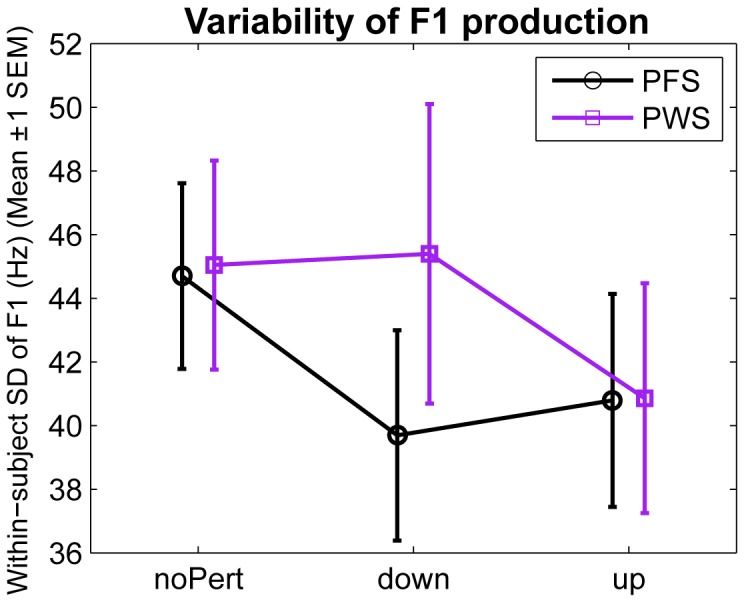
Variability of F1 production in PWS and PFS under the three perturbation conditions. The black and purple curves show mean within-subject, across-trial standard deviations (SDs) of produced F1 in PFS and PWS, respectively. The error bars show ±1 SEM. Notice the lack of significant differences in the SD of produced F1 between groups and between perturbation conditions. This figure shows the results from the limited data set, but similar conclusions can be drawn based on the full data set.

To address the question of whether the compensatory responses to the AF perturbation are more variable on a trial-to-trial basis in PWS than in PFS, we computed the across-trial standard deviation (SD) of the F1 value produced at 300 ms following vowel onset by each subject in each perturbation condition. Figure 5 shows the mean SDs (±1 SEM) in each group as a function of perturbation condition. As can be seen in this figure, the PWS and PFS showed similar F1 SDs, which were not significantly different. This observation was confirmed by a group-level repeated-measures ANOVA with a between-subject factor GROUP (PWS, PFS) and a within-subject factor SHIFT (noPert, Down, Up). The main effect of GROUP did not reach significance (limited data set: F_1,37_ = 0.20, p>0.65; full data set: F_1,37_<1×10^−7^, p>0.99); nor did the main effect of SHIFT (limited data set: F_2,74_ = 1.83; p>0.16; full data set: F_2,74_ = 1.43, p>0.24). The GROUP×SHIFT interaction was also non-significant (limited data set: F_2,47_ = 1.12; p>0.33; full data set: F_2,74_ = 0.052; p>0.95). Therefore there was no evidence that the compensatory response to AF perturbation was more variable in PWS than in PFS.

In rationalizing the weaker-than-normal response in PWS observed above, two possibilities need to be discerned: 1) the response latencies to the online perturbations of AF were longer in PWS than in PFS, and the belated onset of response could have caused the smaller magnitudes of compensation in PWS when comparisons are made on a temporal basis; 2) PWS and PFS had similar response latencies, and the smaller-than-normal compensation magnitudes were due to slower increase of the compensatory changes with time after the response onset. To distinguish these two possibilities, it was necessary to compute the latencies of the participants’ compensatory responses.

There is currently no widely accepted method for computing response latencies to auditory perturbation. In the current study, the latencies of the individual participants’ compensatory responses were computed based on a least-squares two-segment piecewise linear spline fit. The Cohen’s d scores for the Down-Up contrasts were computed as a function of time, which yielded the Down-Up Cohen’s d curve. Briefly, Cohen’s d is a measure of the statistical separation between two sets of random variables. It is defined as the ratio between the difference in the mean values and the composite standard deviation of the two sets of measurements. This approach is based on the assumption that the latency of response is approximately equal under the Down and Up perturbations. We are aware of no theoretical argument or empirical evidence that argues against this assumption.

Obviously, it was meaningful to define response latencies only for subjects who showed significant compensatory responses to the AF perturbation. Here we applied the following criterion for significant compensatory response: the Down-Up Cohen’s d at 300 ms following perturbation onset is greater than 0.3. Under this criterion, 11 of the 21 PWS and 14 of the 18 PFS were judged as compensating significantly when the full data set was analyzed. The ratio of compensating subjects was lower in the PWS (52.4%) than in the PFS (77.8%). However, this between-group difference in percentage was non-significant (p = 0.18, two-tailed Fisher’s exact test). Similar results were found for the limited data set: 15 of the 21 PWS (71%) and 16 of the 18 PFS (89%) were determined as significantly compensating, and the between-group difference in the percentage of non-compensating subjects also did not reach statistical significance (p = 0.25).

Note that this approach of evaluating the existence of compensatory responses in individual participants based on Cohen’s d scores is superior to an alternative, simpler approach based on the absolute magnitudes of the difference between the F1 data from the Down and Up conditions, in that it focuses on the statistical separation between the productions under these two conditions and hence was more robust against spurious fluctuations in the F1 trajectories.

**Figure 6:**
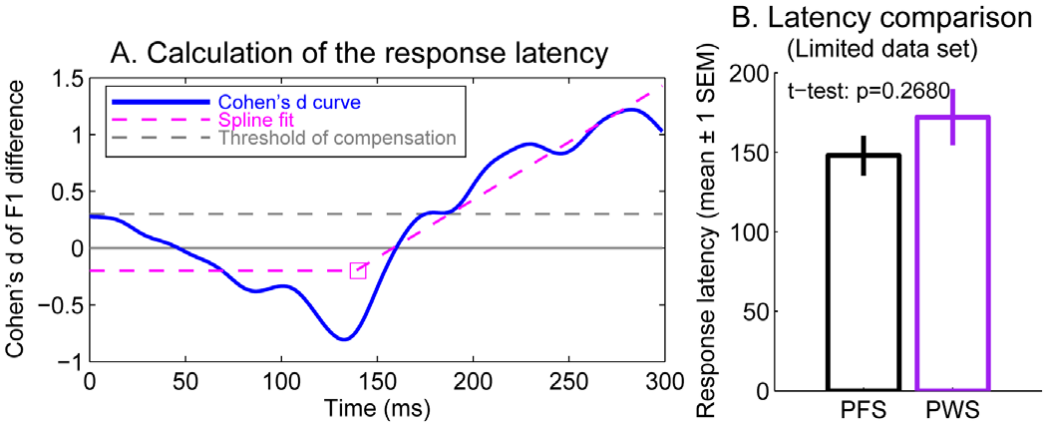
Calculation of the latencies of the compensatory response to the Down and Up perturbations in individual PWS and PFS participants. **A.** An example of the Cohen’s d scores of the differences between the F1 trajectories produced under the Down and Up conditions by an individual subject (blue). A two-segment spline (dashed magenta) was fitted to the Cohen’s d curve. The inflection (break) point of the spline, determined as the response latency of this subject, is shown by the square. The latency of the compensatory response, determined as the zero-crossing time of the fitted spline, is shown by the blue square (see text for details). **B.** Comparison of the group means of the response latencies between the PFS and PWS. These results were obtained from the limited data set (see text for details).

A two-piece linear spline with three adjustable parameters was fitted to the individual participants’ Cohen’s d curves as follows:
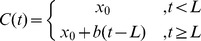



The three adjustable free parameters included 1)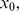 the baseline value before the onset of the perturbation; 2)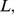 the latency of the response; and 3) 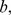 the slope of the linear increase of F1 change with time. The *fmincon* function of the MATLAB Optimization Toolbox was used for the least-square-error fitting. A conservative lower limit of 50 ms was imposed on 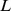 during the optimization, based on the shortest latencies that have been reported in prior studies of pitch and formant perturbation [9], [12], [47], [48]. An example Cohen’s d curve is shown in Fig. 6A, along with the fitted linear spline. The response latency was determined as the value of 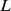 in the resulting fit. Only the limited data set was used in computing the response latencies, because the presence of the cross-adaptation effect in the full data set may lead to under-estimations of the latencies.

We used this more-involved method of fitting a two-segment spline, rather than the simpler approach based on an absolute threshold of Cohen’s d score, because it served to prevent the response magnitude from biasing the calculated latency. If a fixed threshold were used and the time at which the Cohen’s d curve first overcomes this threshold were calculated as the response latency, then the calculated response latencies of the participants with smaller response magnitudes would be longer than those with greater response magnitudes, even if the true onset times of the responses are equal. This is an especially important issue in the current study, because we have observed significant and substantial between-group differences in the magnitudes of the compensatory responses.

Panels Bof Figure 6 shows a comparison of the mean response latencies of the compensating subsets of both groups. The average response latencies were approximately 150–160 ms, and showed no significant between-group difference (p = 0.27, two-tailed two-sample t-test). Therefore the weaker-than-normal compensatory response to the auditory perturbation observed before (Fig. 4) was not attributable to slower onset of the online compensation, but instead was more likely due to a weaker gradual increase in the F1 deviation from the baseline values in the PWS compared to the PFS.

There is evidence that the acuity of the sensory systems can affect the degree to which the motor systems utilize the corresponding sensory feedback for motor control and learning (e.g., [44], [49]). In speech motor control, speakers who have better auditory acuity to vowel formant differences show greater adaptation to the perturbation of AF during the production of the monophthong [ε] [44]. Therefore, the under-compensation we observed in the stuttering participants may be attributable to worse-than-normal auditory acuity for vowel formant (F1) differences. This explanation seemed possible in the light of previous reports of abnormal auditory processing of speech sounds in PWS (e.g., [27], [28], [50], [51]).

**Figure 7:**
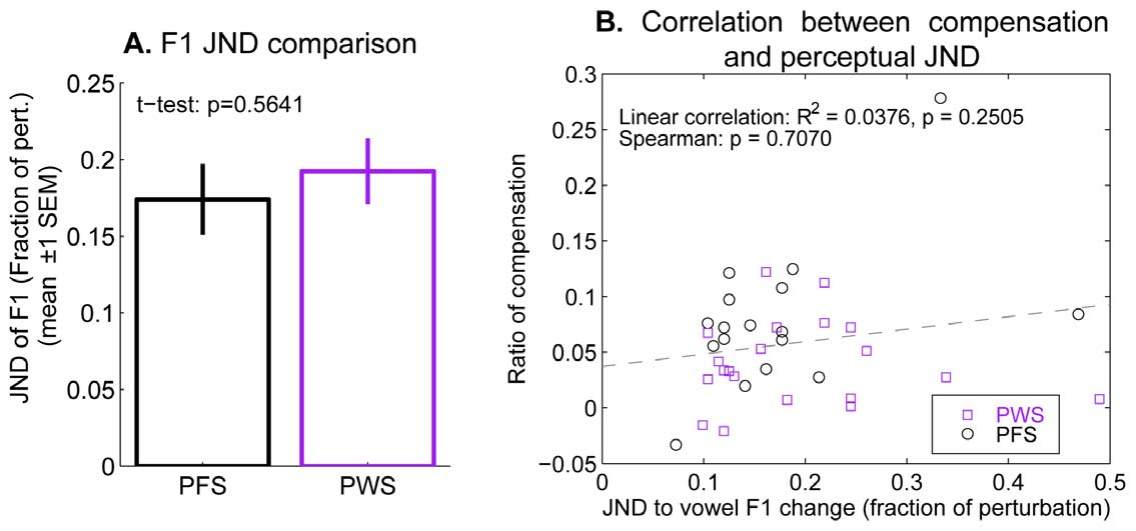
Auditory acuity to differences in F1 of the vowel [ε] and its relation to the magnitude of the compensation to perturbation. **A.** Comparison of the vowel F1 JNDs between the PFS and PWS groups. A fraction of perturbation equal to 1 corresponds to the same magnitude of perturbation as used in the production experiment. **B.** Correlation between the F1 JNDs (abscissa) and the magnitude of the compensation to the AF perturbation (ordinate). Compensation magnitudes from the limited data set analysis are used in this plot. The fraction difference between the F1 produced under the Down and Up conditions, at 300 ms following vowel onset, is used as a measure of compensation magnitude. The black and purple circles show the data from the PFS and PWS participants, respectively.

As described above, we tested this possibility by measuring the participants’ JNDs of F1 of the vowel [ε]. An adaptive staircase procedure (see Methods for details) was used. As Fig. 7A shows, the F1 JND was on average 10.3% higher in the PWS group than in the PFS group, indicating that on average, the PWS participants were slightly worse at detecting F1 differences of the vowel [ε] as compared to PFS. However, this difference was not statistically significant (p = 0.56, two-tailed t-test). Moreover, there was no evidence for systematic cross-participant correlations between their auditory acuity and the magnitude of their compensatory F1 production changes. This held true for the pooled group of PWS and PFS, and for each of the two groups separately (Fig. 7B). These results indicate that PWS’s weaker-than-normal compensation for online perturbations of AF was not the result of an auditory perceptual deficit (i.e., inability to detect the shifts in AF), but instead reflect functional defects in the AF-based online control of speech movements.

## Discussion

In the current study we found that under unanticipated perturbations of AF during the production of monophthongs, participants with persistent developmental stuttering showed online compensatory adjustments to their articulation that were qualitatively similar to the compensatory responses by fluent controls. However, as a group, the magnitudes of the PWS’ compensatory responses were significantly (p<0.05) and substantially (47%) weaker compared to those of the controls, providing evidence for abnormal utilization of AF information by the speech motor system for the control of ongoing movements in developmental stutterers.

Concerning the three hypotheses mentioned in the Introduction, we found no evidence for H1, which posited that PWS had auditory perception deficits that impaired their ability to distinguish effects of the auditory perturbation. The results of our auditory acuity test indicate that the acuity to formant changes around the vowel [ε] was similar in PWS and PFS and not significantly different between the two groups (Fig. 7A).

We also found no evidence for H3, that PWS had increased trial-to-trial variability in their motor responses to auditory errors (Fig. 5). Instead our results supported H2, that PWS have an abnormal gain in their AF control systems for speech. In particular, we found smaller response magnitudes (i.e., a reduced gain) in the AF control system of PWS compared to PFS.

To relate these findings to the existing literature, it is useful to consider the concept of “internal models” in motor control since defects in internal models have often been proposed as possible sources of stuttering (e.g, [52]–[55]). The term ***internal model*** is relatively vague, having several different possible interpretations. Forms of internal model include ***forward models***, which predict sensory consequences from ongoing motor activity, and ***Inverse models***, which are internal models that translate sensory plans (or detected sensory errors) into motor commands. Within the framework of the DIVA (Directions Into Velocities of Articulators) model [32], three main types of inverse model may be involved in speech: (i) an ***auditory-to-motor inverse model*** in the AF control system that translates detected auditory errors into corrective motor commands, (ii) a ***somatosensory-to-motor inverse model*** that translates detected somatosensory errors into corrective motor commands, and (iii) a set of learned ***feedforward motor commands*** that translate desired auditory trajectories into appropriate motor acts without waiting for sensory feedback (hence the term feedforward). In some formulations of speech motor control (e.g., [52], [56]), feedforward motor commands are generated “online” (rather than being read out from memory) using the same auditory-to-motor inverse model used for AF control.

Within this context, our results provide direct evidence for abnormalities in the ***auditory-to-motor inverse model*** of PWS, i.e., deficits in the function of auditory-motor inverse models. A recent study by Loucks et al. [46] reported findings similar to ours, although those investigators used pitch perturbation, instead of formant perturbation. Their findings of significantly smaller-than-normal compensation magnitudes and a non-significant trend toward smaller proportions of compensating responses were consistent with ours. However, whereas Loucks and colleagues reported that the responses of their PWS were delayed with respect to those of their controls, we failed to observe between-group differences in the response latency. This discrepancy needs to be further examined in future studies, with a unified latency calculation algorithm.

The results of the current study and those of [46] are largely consistent with previous hypotheses regarding the role of defective internal models in the mechanisms of stuttering [52], [53], [55], [57]. However, as reviewed in Introduction, these hypotheses were based mostly on somewhat indirect and circumstantial evidence. To our knowledge, the findings of the current study provide the most direct and unequivocal support to date for deficits in internal models of the speech motor system, and in particular indicate possible defects of the auditory-motor inverse mapping in the articulatory control of PWS. Online compensation to the auditory perturbations used in the current study requires the inverse mapping from the auditory space into the space of articulatory movements. For example, under the Up shift of F1, the brain needs to determine the proper counteracting movement, which is an elevation of the jaw and the tongue, because upward errors in F1 of the vowel [ε] are normally caused by lower-than-intended height of the jaw and the tongue body. Deficits in this inverse mapping may cause improper corrective motor commands, which could be manifested as weaker-than-normal compensatory formant changes seen in the current study.

Another possible, and potentially interrelated, contributing factor to the under-compensation in the PWS is inefficient detection of auditory errors due to problems in forward modeling, i.e., the internal prediction of the sensory (auditory in this case) consequences of movement commands. In other words, although from a perceptual point of view, the PWS are not significantly worse than the normal controls in hearing the changes in F1 as suggested by our JND data (Fig. 7A), their auditory-motor interfaces may be not capable of generating error signals and dispatch them to the motor system for gesture corrections as effectively as PFS can under perturbation. To test this possibility and to help pinpoint the detailed mechanisms of the auditory-motor under-compensation, future studies can use the technique of simultaneous electrophysiological recording (e.g., MEG) and auditory-feedback perturbation during speech similar to the approach used by Heinks-Maldonado et al. [58] and examine whether there are any differences in the attenuation of the motor-induced inhibition to self-produced auditory feedback between PWS and normal controls.

In light of a few prior studies, the above-discussed deficit may not be restricted to AF, but may instead be general to the transformations between other sensory modalities and the motor domain (e.g., [59], [60]). For example, Loucks and de Nil [61] reported that PWS showed weaker-than-normal motor adjustments in response to masseter tendon vibration, a manipulation of proprioceptive feedback, during a non-speech jaw movement task. Using an unanticipated mechanical force load to the lower lip during the production of a bilabial stop consonant [p], Caruso and colleagues [62] demonstrated that three PWS participants showed significantly reduced compensations in the EMG activities of the lower lip and significantly longer response latencies compared to three control participants. In another similar study, Bauer et al. [63] reported the preliminary finding that two severe stutterers (out of 10 PWS in that study) failed to compensate for unexpected mechanical perturbation during the production of “sasasar”. Qualitative similarity between these findings and the finding of weakened vowel formant compensation in the current study is intriguing and hints at a general sensory-motor translation deficit. It is possible that certain brain areas that are involved in the integration of multiple modalities of sensory information with ongoing motor control are defective in PWS and this defect may be the common underlying cause for the findings both in the current and above-mentioned studies of sensorimotor control.

For methodological reasons, the current study examined only the fluent speech of PWS. An important question is how these inverse-model deficits may be related to the occurrence of dysfluencies in stuttering, i.e., the primary observable characteristics of this disorder. Regarding this question, there are several possibilities. First, the calculation of local movement corrections for ensuring the successful achievement of speech motor goals in the presence of perturbations or motor variability is but one of the functional roles played by hypothesized inverse models. Another important functional role of these inverse models is the generation of motor commands for the production of syllables and phonemes. On this issue, different researchers seem to attribute different functional roles to inverse models. For example, according to some researchers and models [53]–[55], even in mature adult speakers and for well-learned utterances, inverse models are involved in converting the desired acoustic outcome into the proper speech motor programs. If this is the case, then dysfunction of the inverse models may lead to failures of generating proper motor commands in a timely manner during ongoing speech, which may cause the production process to halt and fall into struggling patterns such as silent blocks, sound prolongations and repetitions.

Other researchers, including authors of the DIVA model [32], [64], [65], hypothesize that the inverse models are primarily responsible for correcting execution errors due to artificial perturbations (as employed by the current study) or natural motor variability and for the learning of speech motor programs (e.g., in children acquiring speech motor skills and adults learning new speech sounds or syllables in foreign languages). However, for well-learned syllables, healthy adult speakers primarily use stored, previously-learned motor programs. In this theoretical framework, a failure to correct for the errors in speech movements may cause error to accumulate and reach a certain threshold where the articulatory process can no longer proceed, manifested as dysfluencies (c.f., [66]). This problem of accumulating error is more serious in longer utterances than in shorter ones, which may account for the observation of the positive correlations between utterance length and the frequency of stuttering [67]–[69]. In addition, the defective inverse models may hinder the proper learning of speech movement programs. This is consistent with the weaker-than-normal cross-trial adaptation of PWS found in the current study (see Fig. 3), an unintended, serendipitous finding that should be examined more carefully in future studies. These insufficiently learned motor programs may generate more articulatory and consequent acoustic and auditory errors, thereby over-taxing inverse internal model-based online error correction mechanisms, which are, unfortunately, impaired in the first place. The formation of such a “vicious cycle” that stems from defective inverse models, seems to be a plausible contributing factor toat least certain types of fluency breakdowns in stuttering.

The aforementioned possible mechanism that involves both internal models and AF-mediated control can be explored further by making specific alterations to the DIVA computational model of speech production. Specifically, we can introduce noise or reduced gain into the inverse model, and observe the effects of such insults on speech motor learning and execution. It should be noted that such a mechanism is different from the AF over-reliance hypothesis [66], in that it does not require an abnormally high relative reliance on or gain of the feedback pathway. In fact, the hypothesis that there may be an abnormally high weight associated with the feedback pathway in stuttering (e.g., as implemented in the simulations of Civier et al. [66]) is inconsistent with the current results. Another shortcoming of the hypothesis that stuttering results from the unstable articulatory behaviors associated with high feedback gain [66] is that many stuttering events take the form of utterance-initial blocks. However, the hypothesis of a weakened feedforward pathway, another key premise of [66], is not incompatible with any aspect of the current findings and could be a viable explanation for utterance-initial blocks. The defective internal model hypothesis does not have this shortcoming, as utterance-initial blocks can be explained by the failure of the inverse models to generate movements for the initial sounds of the utterance.

For simplicity of feedback perturbation and for continuity with previous studies, the current study focused on the static vowel (monophthong) [ε], produced in a prolonged manner and under externally imposed requirements on speaking rate and intensity. Such a setting might have artificially increased the degree of engagement of the feedback pathway in either of both groups of participants, hence potentially limiting the generalizability of the findings to real-life running speech. However, this is not a shortcoming of the current study alone: most previous studies on sensorimotor integration in speech production used highly simplified and regulated “lab speech”. In fact, the duration of the sustained vowel used in the current study (300–500 ms) was shorter than the vowel duration used in previous studies of pitch (e.g., [9], [48], [70]) or formant (e.g., [12]) perturbation, which were typically over 1 s. The relatively short vowel duration rendered it impossible for us to map out the full time course of the evolution of the compensatory responses. Therefore, we cannot rule out the possibility that given longer response time, the compensatory responses of the PWS may approach the normal magnitude. However, a relatively short analysis window (300 ms), close to the average vowel durations in real-life speech, proved to be sufficient for revealing difference in auditory-motor interaction between PWS and controls.

Future studies may employ auditory perturbation techniques during more realistic speech tasks (e.g., oral passage reading), facilitated by online speech recognition techniques [71]. Stuttering is primarily a disorder of the dynamic aspects of speech production, e.g., the sequencing of and transitions between sounds of speech [72]. Most stuttering events occur during multisyllabic, connected speech. How may the findings of the current study be related to the difficulties of achieving proper between-sound and between-syllable transitions in stuttering? Cai et al. [14] showed that AF is utilized by the speech motor system in controlling the magnitude and timing of movements during the between-syllable transitional periods. Specifically, Cai and colleagues showed evidence that AF information from a preceding syllable is used by the speech motor system to help fine-tune spatial aspects of the movements that are necessary for the transition between the end of the preceding syllable and the beginning of the ensuing one. This transitional command calculation may employ, at least in part, the same inverse models as involved in the online error correction. Therefore it is reasonable to speculate that deficits in these inverse models may cause improper transitions between syllables, leading to dysfluencies. Apart from the generation of magnitudes of transitional motor commands, the findings of Cai et al. [14] also support the idea that AF information is used by the speech motor system to fine-tune inter-syllabic timing. In other words, AF also helps to determine the timing of articulatory events. Therefore deficits in AF-motor interaction may cause failures to initiate or terminate syllables at appropriate times. This possibility needs to be addressed by a sensorimotor model of multisyllabic articulation. Such a model does not exist yet. The DIVA model is currently concerned with primarily the production of single syllables. Another approach, the Task Dynamics (TD) model [73] addresses multisyllabic articulation but does not incorporate sensory feedback. The model of Kalveram [74] characterizes the role played by AF explicitly, but is lacking in kinematic details of articulation. The integration of the DIVA model with the GODIVA model [75], a neurocomputational model of the sequencing of syllables in multisyllabic utterances, holds potential for filling this gap and for establishing an appropriate framework for investigating relations between the deficits of auditory-motor interaction and the time-varying aspects of the speech motor system in stuttering.

Persistent stuttering is a neuro-developmental disorder that typically has its onset in early childhood (3–5 years of age). Therefore a thorough understanding of this disorder can only be obtained through investigating the speech motor behaviors in children who stutter. As such, auditory-motor compensation and adaptation in children who stutter and any differences with their normal counterparts [16] are an important topic for future research, especially considering the hypothesized importance for AF in speech motor development [32].
